# Gender, mental health and resilience in armed conflict: listening to life stories of internally displaced women in Colombia

**DOI:** 10.1136/bmjgh-2021-005770

**Published:** 2021-10-07

**Authors:** Emilia Zamora-Moncayo, Rochelle A. Burgess, Laura Fonseca, Mónica González-Gort, Ritsuko Kakuma

**Affiliations:** 1 Escuela de Psicología, Universidad de Las Americas Facultad de Ciencias de la Salud, Quito, Pichincha, Ecuador; 2 Institute for Global Health, UCL, London, UK; 3 Department of Social Work, University of Johannesburg, Auckland Park, South Africa; 4 Facultad de Psicología, Universidad de La Sabana, Chia, Colombia; 5 Department of Psychological and Behavioural Sciences, The London School of Economics and Political Science, London, UK; 6 Centre for Global Mental Health, London School of Hygiene & Tropical Medicine, London, UK

**Keywords:** mental health & psychiatry, public health, qualitative study

## Abstract

For over 60 years, Colombia has endured violent civil conflict forcibly displacing more than 8 million people. Recent efforts have begun to explore mental health consequences of these contexts, with an emphasis on national surveys. To date few Colombian studies explore mental health and well-being from a lived experience perspective. Those that do, overlook processes that enable survival. In response to this gap, we conducted a life history study of seven internally displaced Colombian women in the Cundinamarca department, analysing 18 interview sessions and 36 hours of transcripts. A thematic network analysis, informed by Latin-American perspectives on gender and critical resilience frameworks, explored women’s coping strategies in response to conflict-driven hardships related to mental well-being. Analysis illuminated that: (1) the gendered impacts of the armed conflict on women’s emotional well-being work through exacerbating historical gendered violence and inequality, intensifying existing emotional health challenges, and (2) coping strategies reflect women’s ability to mobilise cognitive, bodied, social, material and symbolic power and resources. Our findings highlight that the sociopolitical contexts of women’s lives are inseparable from their efforts to achieve mental well-being, and the value of deep narrative and historical work to capturing the complexity of women’s experiences within conflict settings. We suggest the importance of social interventions to support the mental health of women in conflict settings, in order to centre the social and political contexts faced by such marginalised groups within efforts to improve mental health.

**Key questions**

**What is already known?**

- Existing evidence highlights the mental health consequences of conflict, and women’s disproportionate burden of emotional distress, particularly in Colombia.
- This evidence is largely drawn from epidemiological studies, overlooking narratives of lived experiences and women’s survival.

**What are the new findings?**

- Women’s resilience and survival in the face of distressing environments are driven through action specifically targeting sociopolitical contexts of their lives.
- Women leverage various forms of power within their survival, embodying Latin-American feminist perspectives on empowerment.

**What do the new findings imply?**

- For women in Colombia and similar conflict-affected settings, mental health responses that foreground social interventions and build on women’s existing strengths are ideal.

## Background

Colombia has been in a state of intermittent internal conflict since the nineteenth century, primarily due to a divided political system and socioeconomic disparity.^1 2^ Approximately 262 619 deaths have been registered to date, and over 8 million people have been forcibly displaced.^3^ Internal displacement in Colombia is unidirectional; individuals are often displaced for life, and potentially for generations.^1^ Displacement as a result of conflict thus represents a multiple loss experience including losses of housing, land, social status, support networks and personal possessions.^1^

Internally displaced people (IDP) face severe danger and adversities in all phases of the displacement process: departure, transit and arrival.^1^ Women are at a higher risk of suffering violence along the pathway of displacement.^4^ These experiences result in psychological distress and greater risks for victimisation, physical ailments and mental disorders.^5^ For example, a qualitative study conducted to explore the health needs of IDP in Colombia, found that as women often become heads of households, delaying their own access to health services was common, as they prioritised their role as caregivers and financial providers over their health, increasing rates of miscarriages and maternal and infant mortality.^6^

Mogollón-Pérez and colleagues^7 8^ also found that mental health and psychosocial impairments were the most common challenges reported by women who were internally displaced in Colombia, across a range of studies. Furthermore, a meta-analysis exploring the burden of common mental disorders (anxiety and depression) and Post-traumatic Stress Disorder (PTSD) among victims of the armed conflict, showed a high prevalence of symptoms, possible cases and confirmed cases of mental health conditions among the IDP.^9 10^ These results are consistent with findings from the recent national mental health survey which reported that 1 in 10 adults in Colombia meet the criteria for a mental disorder.^11^ Findings also confirmed that the presence of mental health impairment is higher in conflict-affected zones and, among women.^11^

Gender-based violence is one of the clearest markers of pre-existing gender inequality and discrimination, and is exacerbated during periods of conflict.^12 13^ In non-conflict scenarios, women already face high risks of sexual abuse and various forms of intimate partner violence (IPV) during their lifetime, with 35% of women experiencing physical and/or sexual violence by an intimate partner or non-partner sexual violence.^14 15^ Evidence shows that in conflict-affected settings this violence escalates significantly leading to serious psychosocial impairments.^16 17^

The complexity of the contexts shaping violence and related mental health outcomes have led many scholars to argue for interventions that centre social challenges. In Colombia, researchers have emphasised the importance of centring individual, family and community needs when planning care and reparation.^18^ Moreover, calls for identification of social and political challenges as the starting point for intervention development is widespread.^19^ Scholars from the global south also suggest the importance of exploring psychological resources; mechanisms or protective factors available to people affected by conflict operate when faced with sociocultural adversity.^18–20^

However, it has been recognised that many victims of conflict-driven forced migration do not develop mental disorders despite being at risk.^21^ Resilience is a concept that has been investigated extensively in recent decades to explore the underlying mechanisms that enable individuals to cope with and overcome adversities.^22^ Resilience frameworks allow us to approach women’s experiences of conflict from a lens that extends beyond victimhood and towards coping strategies. However, existing literature in the Colombian context does not explore resilience and coping among women who were internally displaced. Our work contributes to this gap by addressing the following research question: *how do women’s experiences during conflict illuminate struggle and survival at work in their lives? How does this relate to experiences of distress and opportunities for good mental health and well-being?*

## Methodology

This study was embedded in a larger study exploring the adaptation processes of IDP in Colombia, with the aim to support communities in developing mental health enabling environments,^23^ and was a partnership between Universidad de La Sabana, The Centro Nacional de Memoria Histórica, and the second author (RB). The wider project was a participatory action research (PAR) project, informed by a conceptual framework centring action and engagement to support the study community in projects of change. According to Burgess and colleauges, mental health enabling environments are spaces where the achievement of good mental health is promoted through creating opportunities for communities to reflect on meanings, hopes and desires; and supported action to tackle social issues of importance to their mental health.^24^ Both approaches are anchored within a transformative paradigm,^25^ which views research and evaluation as a route to challenging the status quo, acknowledge limitations within deficit-based paradigms^25^ and seeks study outcomes that change participant lives. We deployed five stages of thinking and acting with communities within the PAR method: *systematising experience*, *collectively analysing and problematising*, *reflecting on and choosing action*, *taking and evaluating action and systematising learning*.^26^ In this paper, we report on data collected as part of the *systematising experience stage*; a substudy focused specifically on life histories of conflict and coping strategies used by women across their journeys of displacement, and its links to emotional distress and well-being.

### Patient and public involvement

The PAR study operated at the level of communities of place, to understand the experiences of *potential* service users in an area, in hopes of improving future service acceptability. Accounts at the heart of this manuscript are owned by women who are potential users of mental health services. Beyond this, PAR principles guiding our work^26^ meant that participants were in the driving seat for much of the research process. Though researchers conceived of the broader research question, methodological choices ensured that study participants had ownership of data produced, through use of open participatory and visual methods, participating in analysis (via member checking activities), and leading data collection within evaluation stages of the research, using photovoice methods (see Burgess and Fonseca^10^ for full details).

### Method: life history

Better understandings of everyday meanings and the complexity of managing distress under adversity enable the design of meaningful policy and interventions.^24^ This is particularly important for the mental health field, as it faces calls to overcome longstanding erasure of lived experiences in favour of the pragmatics of care.^27–30^

To combat this silencing, we used life history (LH) interviews to engage with the intersecting factors that shape experiences of and responses to distress for internally displaced women. LH interviews use questioning to prompt narratives that elaborate on a person’s life experiences in their own words and across their own timelines.^31 32^ Often conducted over multiple sessions with continuous reference to instances of change, LH interviews can explore temporality,^33^ helping to understand how, why and when people move through periods of vulnerability and resilience. This method has been applied in related contexts, including studies of family trajectories through poverty,^33–35^ analysing the impacts of policy on people’s livelihoods^36^ and subjective perspectives and meanings that people ascribe to community experiences.^33^ Furthermore, the LH is a narrative method that has been described as a potential site of transformation for participants, as they come view themselves in new ways that centre their strength and survival in their own stories, and their own words.^37^

### Study site

This study was conducted with women who resettled in a municipality of Colombia located in the Department of Cundinamarca, Sabana Centro Province. This site (Name withheld to ensure participant anonymity.) has experienced large economic growth in the past 10 years, supported primarily by the floriculturist and construction sectors. This site leads the department in Gross Domestic Product (GDP) participation with 6.06% and is the second with the highest degree of municipal economic importance. Recent studies cite this as the primary draw to this area^10^ and approximately 32% of the IDP who arrive in the province, settle near there.^38 39^

### Sample and recruitment

Participants in the wider study were randomly selected from the municipality’s official register of displaced victims from different conflict-affected zones in Colombia. Seven women agreed to participate in the LH substudy. At the time of the interviews, participants had been living at the study site between 3 and 10 years. Concerns over sample size were alleviated through the repeated measures approach of LHs, and the depth and quality of the dialogue in interviews,^40^ which helped saturate the themes and concepts relevant to the larger study. Online supplemental table 1 provides additional participant information.

10.1136/bmjgh-2021-005770.supp1 Supplementary data

### Data collection

The interview guide was informed by preliminary findings from the wider study,^10^ developed by RB, and structured around history (including childhood), adulthood (with an emphasis on birth of their own children) and life in their new home (see section 1 of online supplemental data). Interviews were completed by MG-G and another MSc student, and supervised by LF and RB between August and October 2017. Participants were contacted via telephone to schedule three different interview sessions.^41^ Interviews lasted between 1 and 3 hours. Eighteen interview sessions were completed and a total of 36 hours of conversation transcribed verbatim by the study team.

### Conceptual framework

Following initial readings and preliminary coding of transcripts, a discussion between the analysis team was completed. We identified two broad directions of the data; descriptions of factors driving distress, and survival strategies. We opted to maintain an analytical focus on drivers of distress, AND coping strategies for two reasons. First, LH and narrative theory argue for the preservation of contexts in making sense of people’s experiences and practices.^42^ To separate these aspects into multiple papers or analyses would be to sever the narrative coherence of women’s stories. Furthermore, as we view drivers of distress to be in a natural dialogue with coping strategies in real-world settings, this would emerge as what Tsoukas^43^ defines as a false separation of context from action for the purpose of simplifying analytical processes. Second, maintaining both dimensions in our analysis also responds to calls to embrace complexity in health service research, which requires theorising that generates rich pictures of complex phenomena, values descriptive detail, narrative coherence and focus on real-world action, to better understand macro social factors and their local manifestations in people’s lives.^44^

Our analysis of drivers of women’s distress was fully data driven. Our analysis of coping was guided by two conceptual frameworks. Despite recognition of women’s struggles in conflict, very little literature explores women’s own narratives of their distress, or their survival and agency in the context of conflict. As such, we applied a Latin-American feminist perspective to frame our understandings of women’s accounts of coping and survival. Communitarian feminism originates in indigenous women’s efforts to decolonise views of feminists as a ‘white, middle-class heterosexual women’.^45 46^ According to the framework, Latin-American women’s struggles cannot be understood from the individualistic positions that dominate western feminism, as it separates women from the community and territory.^45^ Communitarian feminists argue that non-hegemonic knowledge present within social practices underpinning communal organisation are critical to recognising the empowerment and agency within women’s stories, even within ongoing contexts of oppression.^46^ This decision also aligned with the conceptual framework of the wider PAR study.

Our second analytical framework was informed by the wider study’s interest in elevating narratives of everyday survival within conflict and reconstruction settings, to counter western hegemonic ideas of individual resilience which lead to victim blame^47^ or obscure political and social dynamics of life.^48^ For women this is particularly important, given that prolonged displacement and continued adversity have been shown to negatively impact on survival.^49^ Other critical scholars argue that the dominance of resilience research in populations from the Global North do not appropriately account for cultural specificities of the Global South that frame capacities for resilience.^50–52^ As such, we applied Skovdal and Daniel’s^50^ resilience framework that views capacities for resilience as embedded to political economy, community-based networks and the households where everyday coping strategies create pathways to resilience.^50^ Analysing women’s stories through these lenses allowed us to disrupt the dominant paradigm of mental health sciences that has been charged with erasure and simplification of women’s lived experiences.^29^

### Data analysis

Interviews were analysed in their original language. Extracts for team analysis and content selected for inclusion in the report was translated to English. In addition, specific quotes were discussed with three independent native Spanish-speaking researchers to verify the accuracy of the interpretation.^53^ Analysis applied thematic network analysis, involving an iterative process with multiple readings of the data and refining of themes and networks.^54^ NVivo V.12 software was used to help organise data. After the first reading of half the transcripts, an initial coding framework parsed the data into two broad categories: experiences of distress and survival strategies. The initial framework that included broad thematic categories linked to drivers and coping (ie, violence, pregnancy, survival) was devised by EZ-M and discussed with RB and RK.

**Figure 1:**
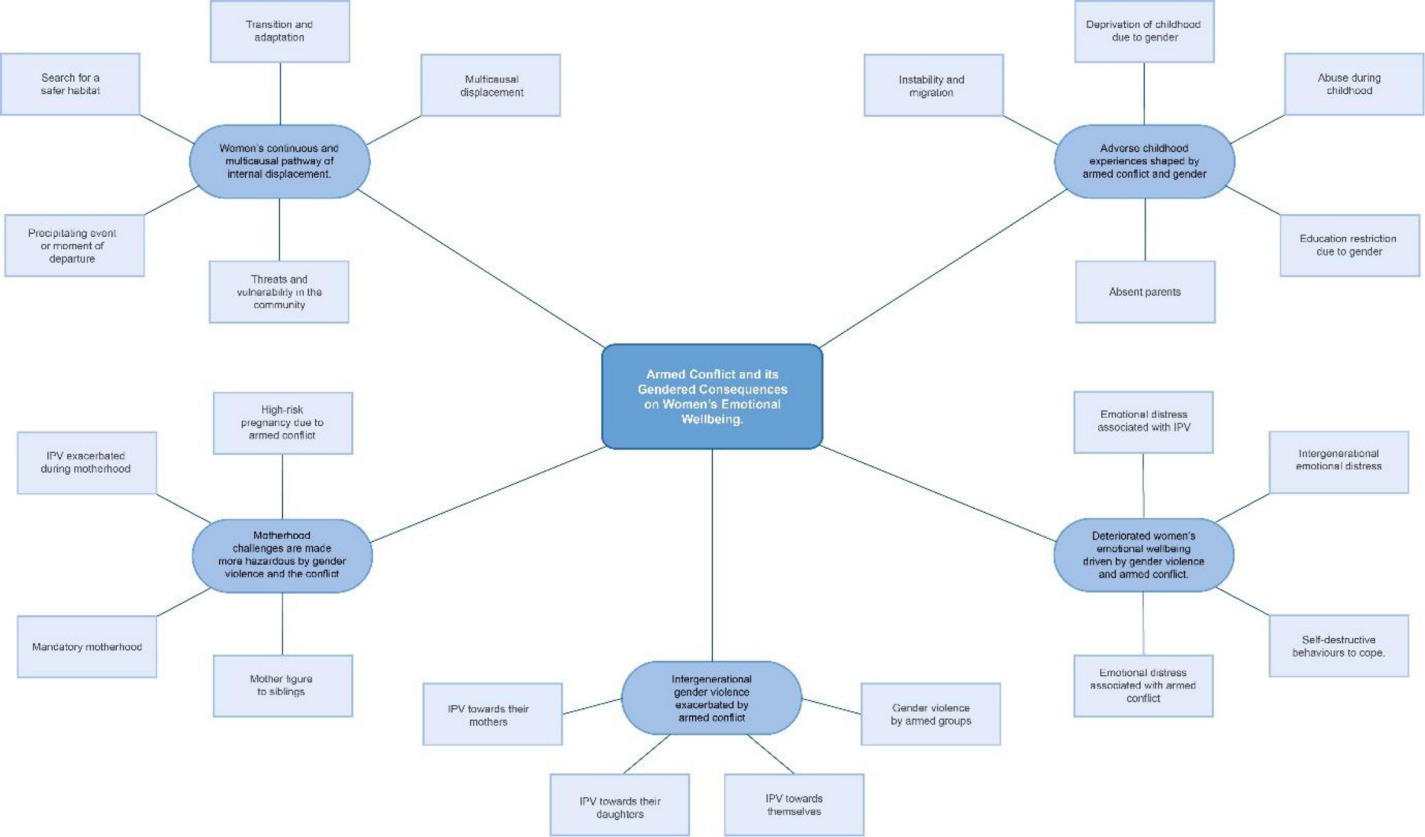
Global theme 1: armed conflict and its gendered consequences on women’s emotional Well-being

**Figure 2:**
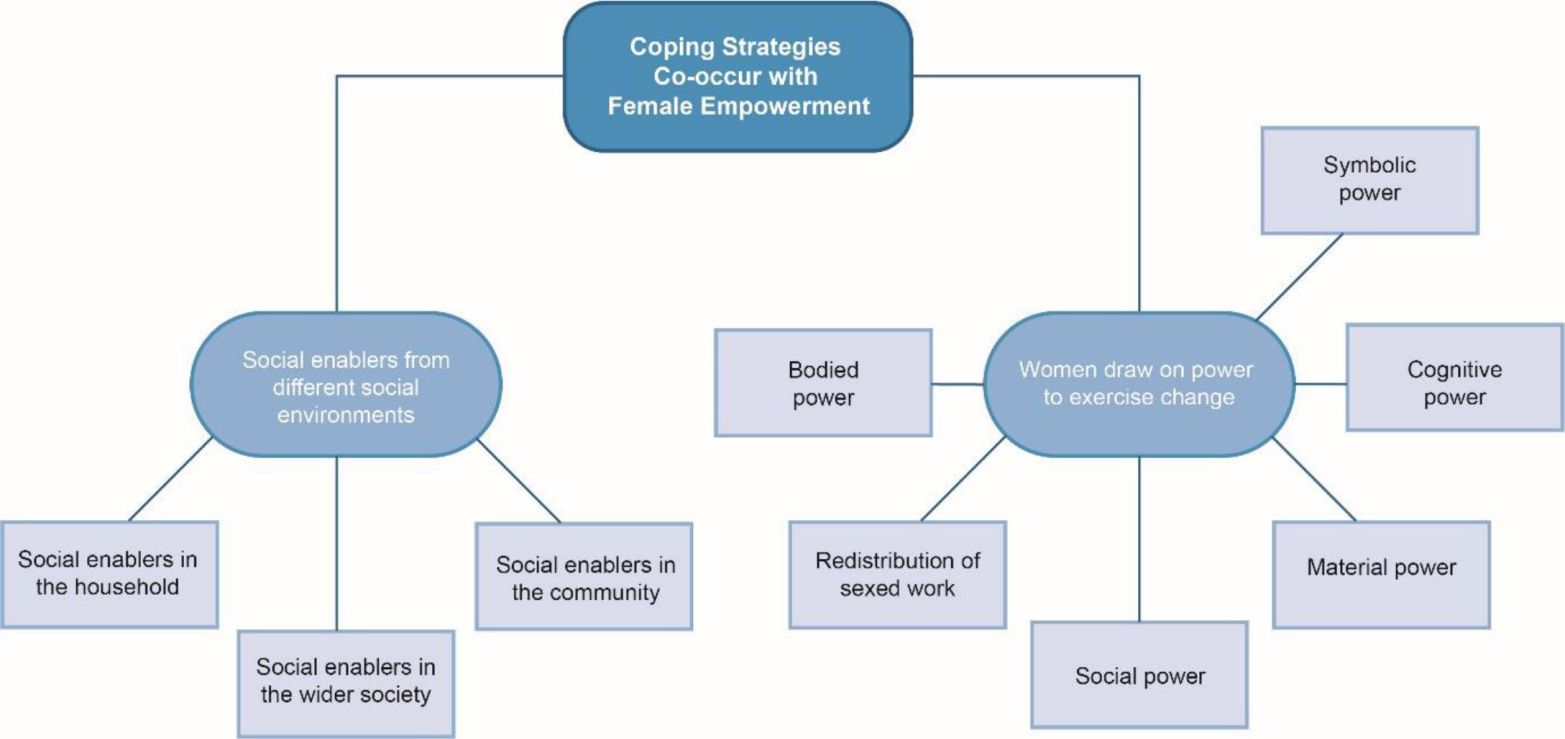
Coping strategies co-occur with female empowerment.

The full sample was then revisited using the coding framework to develop more detailed basic and organising themes in line with Attride-Stirling’s process.^54^ Saturation was determined when identification of new themes ended. Basic themes were then grouped into seven organising themes. Organising themes were reviewed, paying close attention to context, and structured into two global themes. Global themes are depicted as web-like nets to remove any notion of hierarchy and emphasising the interconnectivity of themes.^54^ See figures 1 and 2, along with a full coding framework (see online supplemental data) for full details of the analysis.

## Findings

Our global themes describe women’s realities, the conflict’s relationship to mental health and well-being and survival. Pseudonyms are used throughout and some details have been changed in quotes where required to ensure anonymity.

### Global theme 1: armed conflict and its gendered consequences on women’s emotional well-being

This thematic network explores how women’s life stories were shaped by the gendered nature of armed conflict. Four organising themes illuminate experiences which were identified as leading to the deterioration of women’s mental health and emotional well-being: (1) adverse childhood experiences shaped by conflict and gender; (2) multicausal and continuous pathways of displacement and (3) challenging experiences during motherhood created by the conflict.

#### Adverse childhood experiences shaped by armed conflict and gender

Women’s childhood stories reflected adverse experiences driven by the armed conflict, socioeconomic status and gender starting early in life. For instance, a father’s absence or abandonment due to conflict-related violence was common in nearly every woman’s life story. This was directly linked to experiences of distress by their mothers, who had assumed sole responsibility of raising the children and financially supporting the household:

Well, my mom had eight children and sadly I was the 5th, and I didn’t have my father’s support. [….] My mother practically raised us all by herself. She suffered a lot, poor her. (María)

My mom had it really hard. Imagine, with the minimum wage having to pay rent, do grocery shopping, school, uniforms, school supplies, everything. […] I don’t know how my mom managed to make ends meet. (Dora)

Other adverse experiences such as physical and psychological abuse perpetrated by their mothers as forms of discipline were described as a common practice. When asked about the disciplinary practices used by their mothers, Maria suggested a relationship between the mother–daughter bond and the severity of the physical abuse:

Sometimes she was very rough, sometimes we would cross the line […] and she hit us [hard], she was very rough, mostly with me and the third [referring to the third sibling]. […] She would hit harder the ones who had more of a bond with her. (María)

Adverse experiences during childhood and adolescence were also shaped by an oppressive and exploitative patriarchy. The conflict necessitated that as older women took over men’s roles in managing the house, younger women stepped in to fill in their mothers absence. A recurring theme throughout the interviews was the interruption of studies to do housework and care for siblings. This emerged at very early ages and it was recognised by some women as a distressing experience that deprived them of their childhood:

Many times, I couldn’t go school because there was no one to look after my siblings […] it was hard […] I had to take the responsibility of taking care of my brother, and that made me a bit angry, because a [12-year old] child doesn’t understand […] She [her mother] says that she is sorry because I had to lose my childhood to commit to my siblings, to take care of them, to do things that she would do. (Dora)

Many participantslived through displacement by the conflict since early on in their lives. For these participants, it was clear how conflict disrupted typical developmental milestones and experiences. *Rigoberta* was 7 years old when her family was forcibly displaced, and the resulting interruption to education was a difficult experience shaping her childhood:

I was very young, I barely understood things […] I saw they would run from one place to another[…], yes, it was hard […] I had to switch schools and I was very behind […]. There were many times where they took me out of school because of that [the armed conflict] so I had to retake the same school year again and again. (Rigoberta)

Participants also reported experiencing abuse from members of armed groups including objectification and sexualisation of women’s bodies, even during childhood. Their sharing of these experiences revealed long histories of emotional distress:

One time a group of men came at like 9.30pm […] they told my aunt: ‘could we borrow the girls, we are having a party’, and we were already sleeping but my aunt said: ‘go, go, what if they shoot the house down’. At that time everyone had one or two guns. […] When we arrived, we were seven women dancing with so many men. […] I was getting increasingly nervous […] I looked at one of the corners of the place and there was an [big] armament. I started to cry, asking if we could leave […] we left, but that was so scary, what fear, what a terror I felt. (María)

#### Women’s internal displacement pathway is continuous and multicausal

Women’s narratives of internal displacement revealed multicausal and continuous pathways without a culmination point. Participants described multiple causes for displacement associated to conflict, at different times in their lives:

Because of the conflict we had to flee Valledupar, then we left Barranquilla, then la Guajira, later Zipaquirá, and now I came by myself here [site’s name]. (Sara)

We grew up in San Jacinto, we were displaced by the armed conflict, my childhood has hard, my mom felt obligated to sell the house for very little, we went to my grandparents’ house. (Lucía)

To fully appreciate the complexity of displacement, we explore Maria’s narrative in detail, who serves as an emblematic case that illuminates threads shared by all the women in our sample.

Maria’s first displacement revolved involved fleeing her home in fear of being pulled into sex trafficking. Her experience highlights the shared theme of women’s bodies as spaces where power relations characterised by objectification and repression were interwoven throughout the conflict experience.

… At that time there were these trucks that would pick up 5 or 10 men, and there they would make misdeeds [sexual activity], so they were looking for my sister and I […] We had to leave to another town. (María)

María’s second forced displacement was also common within our sample, and one of the most reported causes of women’s displacement in the country,^55 56^ namely her partner being threatened by the guerrillas. This narrative of displacement reflects the intersectional violences linked to the conflict, where social, financial and human capital losses compound and intersect.

They told him to leave because they [the guerrillas] were looking for all the workers […] to kill them, so he was practically threatened. It was horrible, you were walking, and you would have the feeling that someone was going to shoot you from behind […] We lost so much. (María)

Stories of displacement also intersected with familial issues that reflect other complex social problems. For instance, María described that her third displacement was to avoid family conflict related to drug selling. Drug cartels boomed during the most difficult years of the conflict, leading to many vulnerable groups becoming wrapped up in the production, sale and trafficking of drugs:

We left [town’s name] again because [her husband’s] older daughter married a shit man, and we had to leave, so technically we were displaced twice but not for the same reason, this time it was to avoid problems with my son in law[…] he was a drug dealer. (Maria)

#### Challenges with motherhood are made more hazardous by intersecting violence(s)

As noted by Dora earlier, most of the women in our sample linked their first motherhood experiences to taking on the caregiver role for siblings in the absence of parents during childhood. This requirement was part of a wider acknowledgement motherhood as an inescapable obligation, as further supported below:

If one is getting married at 30 years old, one has to have a child immediately. (María)

Well yes, it is a blessing [having children], because some of them would come to cheer up the home. It was hard for me [motherhood], *but I had to*. (Sara, emphasis added)

This ‘mandatory motherhood’ (maternidad obligatoria) is described by communitarian feminists, as the recognition that motherhood is inherent to women’s nature, restricting their agency on their bodies. Women also described experiences of unwanted pregnancies as unpleasant, and consistently associated with the exacerbation of gender violence. Some women reported being abandoned by their partners, having to take all the responsibility of the childcare and the household:

I became pregnant, we took the decision of having the child, but let’s say that he didn’t care about that period (cries), […] he decided to do what many men do, don’t care about anything. (Rigoberta)

The armed conflict had direct consequences on their experience of motherhood. In addition to the distress linked to forced migration with their children after the loss of their partners, women identified emotional distress linked to conflict and displacement affecting their pregnancies. Dora, who experienced five displacements notes:

[…] the stress and everything I lived […] affects one’s pregnancy a lot. I had contractions and I was all the time at the E.R. They put me on medication to keep [the baby] because I wanted him to be born. (Dora)

Or, María, who shared a story of a family member who lost a pregnancy because of exposure to violence:

Horrible! [referring to the pregnancy] I almost lost the pregnancy when I was three months pregnant. […] My sister did lose her child […] she saw that they killed a man in front of her house, and she filled with nerves.

Armed conflict was only one form of violence linked to deterioration of emotional well-being. Structural violence (economic insecurity), IPV and family drug abuse were identified as causes of their distress. For example, participants highlighted that emotional distress experienced by women in their families impacted on their well-being, as noted by Josefina, who experienced IPV linked to the conflict, and drug use, during different stages of her life:

The father of my child called me, he told me that he accepted me with the child, and that he was going to change, and I, as a fool, went back to him […] he almost killed me from a beating. I didn’t report him because I was so afraid of him, I spent 3 days hospitalized […] I remember telling him that I had my parent’s support and that I could leave anytime and that’s when the problems started.

With all the problems that I had, I distract[ed] myself. […] Sometimes I solved the problems by drinking. I didn’t like arriving home, One time because I was so tired, I took like 30 pills. It was hard, and the worst part was that my two brothers [consumed drugs] too, because my mom was never able to stop doing drugs, for her it was very hard to quit. That is why she took her life away. (Josefina)

### Global theme 2: responding to the emotional consequences of violence—coping strategies and female empowerment

Our second network explores women’s coping strategies that respond to the challenges described in the previous section. Two broad survival processes were drawn out from women’s stories (1) efforts to draw on social enablers in wider environments and (2) drawing on forms of power to mobilise for change.

#### Social enablers from different social environments

Our analysis suggests that women’s social enablement drew on social and emotional resources provided at one of the following levels: (1) the household, which involved drawing on family members emotional support and physical assets and resources; (2) the community, which refers to transformative social spaces and relationships outside the family and (3) ‘wider society’, which refers to the opportunities that government provision of health and welfare services offer people to cope with hardship, or political economy.^50^

For example, women described the importance of accessing material and relational resources within their household. Family support, especially from female members, during pregnancies, early motherhood and when experiencing IPV was considered a key resource to enable survival:

He told me to get an abortion, I had already 2 months of pregnancy, and he told me to get an abortion……I was so afraid of him so I told him that I was going to do it […] but my parents told me that I was not the first woman to be by herself with her child, and they told me they were going to support me…. So, I felt a support, I didn’t feel alone (Josefina)

Participants explicitly described the importance of these material resources as part of the settlement process following displacement. This included housing, or income:

[…] I first arrived to my sister’s house, to lean on her […] We [her daughter and herself] lived there for about 6 months. (Rigoberta)

Women referred to the fracturing of families due to displacement as a difficult experience, noting the value that family connection and support has in their lives and the role this played in resilience.

[…]It could have been different, one living there, we would be close to our family, they would be so helpful, his family and mine, like everything would be different, it’s hard, this is also hard, very hard. (Miriam)

Relationships with neighbours or other members in their communities of place were identified as important sources of social support and solidarity, providing specific forms of social enablement. For example, women described how access to relational and material resources helped them to cope with adversity by providing connections to basic amenities such as electricity, or employment opportunities. The below quotes exemplify the positive potential of these community relationships:

The house didn’t have electricity anymore, so the mam [neighbour] gave us electricity through a cable, and [we] put the light bulb inside the little room where we used to sleep […] They are good neighbours […] they are there for anything […] I would run into them at the park or anywhere, […] and when I saw them […] my heart would fill with joy. (Sara)

She [her neighbour and friend] told me: ‘Let’s go to [site’s name], my brother needs someone to work at the farm’, I told her I didn’t have the money, she told me ‘don’t be silly, I’ll lend you the money, but let’s go’. (Rigoberta)

Women also highlighted several forms of government aid that enabled their management of hardship. The IDP status was identified as an enabler, allowing them to access resources. This governmental recognition is part of measures taken by the Colombian state to compensate the victims for the losses and consequences of the armed conflict.^55^ Women also identified economic aid, maternity programmes, housing restitution, and preferential access to training and technical education as some of the opportunities that enabled coping:

Well my mom received the ‘family-nation’ subsidy, and with that she provided for my brothers […] The family-nation is a help that one receives every two months, for studies, growth and development […] I’ve been receiving it for two years. (Josefina)

So, after I finished [to validate] my high school diploma, I started to study the technical training on children’s education and psychology, that is why I work now. (Rigoberta)

We go a lot to the park or to the library […] she [her daughter] loves to read, and they have a lot of didactic games. […] we didn’t have parks for children and all those things […] That joy, for example, of taking the children to the library, or that she can go to school in peace, they don’t have to live all those things that we had to. (Lucía)

However, implementation of these polices was labelled as slow and incomplete. As Sara notes

We receive very little help, the economic reparation hasn’t arrived […] it has been 17 years, we’ll have to wait and see.

#### Drawing on power to exercise change

Our positionalities as feminist and critical scholars, with an interest in illuminating the agency of oppressed persons, drove a desire to explicitly explore how power was at work within women’s coping strategies. Some women spoke of doing things independently or ‘solita’, however, they did not explicitly identify or label themselves, or their individual and collective acts of perseverance as a form of power. Critical scholars such as Paulo Freire^55^ and Frantz Fanon^57^ have noted this as reflecting processes of internalised oppression, where marginalised actors internalise negative self-scripts, or overlook the strengths embodied in their everyday acts of survival. The Latin-American feminist framework described previously enabled a reflection on collective modalities of power, which further embeds our discussions of coping within a more indigenous system of knowledge and praxis. Coping strategies reflected five forms of power discussed in the work of Latin-American feminists^58–60^: *cognitive power*, *bodied power*, *social power*, *material power and symbolic power*. We also noted a redistribution of gendered work, linked to Rubio’s analysis of female empowerment in Latin America.^46^

Cognitive power consists of the acquisition of knowledge and wisdom that contributes to a process of liberation from structures that limit women’s social, intellectual and political participation.^46^ Women in our study specifically referred to education as an opportunity to improve their sense of self-worth and participate in the economic market. Training courses were also described as enjoyable experiences.

I would like to do something productive, that makes me feel like I am doing something with my life [….] I would like [to study] teaching or children education, and recently I’ve been wanting to start to study English, I am now looking to get a scholarship to go to study far abroad. (Lucía)

We are currently taking two courses, gastronomy and entrepreneurship. It is very cool. (María)

Bodied power includes decisions taken by women to boost their bodies’ joy, pleasure and vitality,^46^ and also involves rejection of oppression and violence towards females’ bodies by others.^46^ Women mentioned many circumstances in which they exercised bodied power to face the violence perpetrated by their intimate partners.

I arrived that evening and started packing. He asked me what has happened. I answered fiercely: ‘Well I’m leaving, I’m leaving you here. I can leave whenever I want. Don’t you see I already have my things packed’. I told him that. I bought the ticket at 8pm and left. (Rigoberta)

Social power is defined as the development and strengthening of friendships and social networks that bridge individual and community forms of empowerment, with the potential to achieve wider social change.^46^ In our sample, the exertion of bodied power by women often overlapped with social power, as the family and the community held wider roles in supporting women’s decisions to breaking cycles of IPV.

And my mother always supported me, she always loved my child. When I told her, I was going to split up [with my husband], she told me that it was fine, that I should do it, so I took the decision, and If I had to be alone, well I’d stay with my child, but living a life like that [suffering physical IPV] No! So, I split up. (Josefina)

Women in our sample also supported other women facing abandonment or gender violence, highlighting the bidirectional and gendered nature of social power:

She is very nice [a friend], she watched over me when I was pregnant […] she was like: ‘what are you doing there all alone and bored, come here so that we can chat’ […] We would cook together, I taught her what I know […] and yes, she also teaches me to do things. (Dora)

That man [her brother in law] left to work in [site’s name] and my sister was suffering a lot. He would come back and beat her up, and so I told her to come live with me […] She came, brought her children and she put them at school. (Sofia)

Access to and control of natural and economic resources reflects material power.^46^ Women’s experiences of earning money varied. Some spoke about leaving formal work when they became pregnant, and some never returned after giving birth to their first child.

What I liked was to work and live well. But not in the kitchen. (Sara)

I stopped working when I became pregnant […] I couldn’t do it with my belly, so I didn’t work again. (Josefina)

Five of the seven women reported engaging in informal commercial activities throughout their lives, typically selling food or cosmetic products. This was particularly the case for women without partners:

My brother-in-law started to bring avocados […] so, I would take a little basket and sell the avocados. (Rigoberta)

However, women including Miriam, spoke about having to accept poor working conditions linked to precarious employment due to the lack of other opportunities and necessity for economic means:

Sometimes one gets sick. At least me, I got all these spots from working there, too many chemicals, sometimes I’m cutting and fumigating, and I can’t even leave the block, I get allergies, but I have to do it, [….] there’s nothing else to do. (Miriam)

Women disliked being economically dependent on their partners, and so a recurrent theme in the interviews was that work was a source of self-realisation—connecting cognitive power to the decision to seek economic independence.

I don’t like doing nothing, I don’t like him to support me financially. So, [when I was working] I felt like I was finally helping. (Dora)

Women referred to religion as a source of spirituality and support during the displacement process, as well as during motherhood, which has been argued as a source of cognitive and social power:

My beautiful God is the one who has helped my children, my family and me to move forward. (Sara)

However, complexity of drawing on this power source was identified, given that religion was referred to as also reinforcing women’s oppression by promoting more traditional gender roles. As noted by Maria: ‘*The Bible gives way more emphasis and importance to the father…the father is the one that is supposed to guarantee and sustain the home*’.

#### Redistribution of gendered work

Redistribution of gendered work, or ‘the care of life’ (el cuidado de la vida) has been described as critical to female empowerment.^46^ We identified several instances in all women’s stories where they challenged gender in order to overcome limiting social, economic contexts they faced.

I do nothing on Sundays, I don’t cook, don’t shower the kids, he has to do everything. (Lucía)

One has to teach them [men] [to do the house chores] since they are little kids […] Men have to help! But if one doesn’t teach them, they get used to being macho, to not help. (Dora)

When asked about how their lives would have been without the conflict, one participant suggested a positive outcome of migration—it released them from their housework and childcare responsibilities, and gave them the chance o become the household providers, for emancipation in some regards:

Maybe if I stayed there, I would have been the typical ‘I’m the one that does everything’ wife, I’m the one that does the ironing, the washing. [instead] I go here and there. Yes, maybe it would have been a sedentary life. (Rigoberta)

## Discussion

Through an analysis of detailed LHs, this study highlighted the gendered and temporal dynamics of drivers of emotional distress faced by a group of women internally displaced by the armed conflict. However, in exploring women’s non-hegemonic and embodied knowledge through regional feminist and critical resilience frameworks, the power mobilised within women’s everyday strategies for survival were centred. In the face of complex forms of social oppression deepened by conflict-related factors, women offered clear resistance to their victimisation, which has meaningful lessons for how we work to promote their mental health and well-being.

Our findings align with others in the country which suggest the permanence of displacement.^1^ However, we also note that displacement is multicausal and continuous; women noted multiple migrations across their lifetimes. This often linked to specific gendered vulnerabilities; threats of violence to them, or male heads of households. This continual instability had impacts on their emotional well-being, deepening distress established by other structural contexts and challenges. To our knowledge, our study is the first to identify this take and suggests the need for further work in this area.

Themes identified as drivers of distress by women are also shared with other patriarchal societies that do not experience active conflict.^61^ However, the gendered physical, structural and intergenerational dynamics of their lives were exacerbated by the dynamics of conflict. Women’s accounts of sexual harassment and abuse perpetrated by armed combatants resonate with literature documenting high rates of sexual violence in conflict-affected zones.^62 63^ Many traditional responsibilities forced on women are compounded during conflict—an outcome reported by all our participants. For communitarian feminists, this implies exploitation of women’s unpaid labour, that privileges men with more free time, greater income and opportunities for sociopolitical representation and power.^58^ In the Colombian context, it could be argued that enduring conflict is partially enabled by a culturally embedded knowledge and awareness that women will step in and fulfil the absence of men in the household.

The burdens of motherhood on mental health are well known. However, we highlighted the emotional distress connected to the burden of *mandatory motherhood*, that continues under the strain of conflict and displacement. Latin-American feminists argue that motherhood is not a choice for several reasons: (1)abortion in most Latin-American countries is penalised; (2) sex education programmes lack a comprehensive approach, contributing to unwanted and teenage pregnancies and (3) women’s lives are validated only through motherhood roles,^60 64–66^ the latter particularly resonated with women in our sample.

Beyond the distress caused by conflict and structural violence, women also noted the impact that familial mental health challenges had on their own mental well-being. This suggests the importance of intergenerational dynamics to mental health in this context. This could be shaped by interlinking phenomena. First, that the entrenched conflict across generations, means that generations of families would experience similar challenges, with similar emotional consequences. The second, points to the intergenerational nature of trauma itself, which has been associated to mental health in families, particularly around how traumatic experiences of the mother influence the mental health of children later in life.^67–69^ For example Giladi and Bell^67^ argue that families affected by historical traumas can display residual effects of distress and emotional and psychosocial disorders even three generations after the traumatic events. The majority of literature in this area explores cultural and historical traumas in populations of holocaust survivors and indigenous peoples.^68^ Evidence suggests that traumatic effects can become embedded in collective, cultural memory and passed on by the same mechanisms through which culture itself is transmitted.^70^ Given the importance of family to women in this study and to processes of mental health recovery more widely^10^ it is worth exploring these dynamics in future studies.

Women’s accounts highlighted their ability to leverage various forms of power in overcoming adversity. Critically, women did not identify or describe themselves as ‘powerful’. Our analysis sought to counter this epistemic self-silencing, through explicitly mapping their actions onto a framework and paradigm sensitive to women’s power at work. In doing so, we illuminated the complexity of power and oppression in their lives and disrupt the dominant narrative that focuses on deficits and victimhood of women in conflict. For example, while armed conflict reinforced an oppressive patriarchy, it simultaneously created opportunities for women to progress in some areas of their economic and social lives. Examples included opportunities for participation in the market economy and strengthening informal networks which resulted from the exercise of their material and social power. This aligns with researchers in the wider field of conflict studies, who note that these gains are only possible given the fluctuation of gender roles that occur in the time of conflict.^71^

Furthermore, while all women noted the importance of formal education in their lives as key to their change, they did not identify their own cultural or embodied knowledges as meaningful to, or contributing to their processes of change and action. This form of critical consciousness about one’s capacity to wield individual power is crucial to change, but its importance is often under acknowledged. Black feminist and other subaltern scholarship, such as Audre Lorde, note that women’s selves are often hidden from them through a lack of opportunity to encounter indigenous knowledges.^72^ For example, recent work with marginalised women exposed to everyday violence(s) in South Africa highlighted the ability of narrative interventions which re-story women’s lives through reflecting on their strengths, within cultural framings of wellness (Ubuntu principles) significantly reduced symptoms of depression.^73^ Pathways to developing a more critical awareness of women’s bodied and cultural power within the spaces of interventions are worth exploring within future studies.

There are natural limits to resilience in the face of the entrenched systemic social challenges women face. This has important implications for the nature of women’s mental health interventions, as traditional approaches tend to overlook the relationship between personal distress and social oppression. In our previous work with IDP communities, we proposed specific pathways to enable mental health promotion and recovery in Colombia (see Burgess and Fonseca^10^). In Table 1, we revisit these recommendations with specific suggestions for women affected by conflict, anchored to a competency approach that builds on women’s existing strengths and efforts to tackle problematic social contexts in their lives, as the thrust of treatment and support aims.

**Table 1:** Proposed social interventions to address contexts of women’s mental distress in conflict and promote mental health competencies

Community mental health competencies (Burgess 2012^78^; Burgess *et al* 2017^23^)	Local definition and contexts tackled	Sample social interventions and strategies to develop competency
**Knowledge**	**Knowledge about mental health-related services**, when they should be accessed (including support with substance use) **Promoting knowledge about indigenous practices and systems of healing; and organisations that work with survivors of IPV** (*Cultural and Political contexts*)	Group-based narrative therapy, combined with social interventions/support^72^
**Safe spaces and dialogue**	**Spaces to promote the development of critical consciousness** to explore how structural issues, particularly the patriarchy, link to mental health experiences Work to **challenge wider public discourses** that enable gender-based violence (*Cultural and political contexts*)	Community conversations Emphasis on intergenerational approach (conversations between women and their female lineage) Community radio and other media projects (film, storytelling, performance art, photography)^79–81^
**Solidarity and identification of local strengths**	**Spaces to identify and build on local strengths and existing capacities** in order to establish long-term local efforts to deal with structural violence (*Cultural Economic and political contexts*)	Group-based approaches such as women’s circles which are augmented with microfinance or livelihood projects^82^
**Partnerships with external agencies**	**Opportunities to establish links to public and private sector agents** to support efforts to ensure stable economic and social development (*Economic, and political contexts*)	Training for victim support advisor to support establishing networks between social and economic organisations and communities Appropriate funding mechanisms for women’s development programming

IPV, intimate partner violence.

Our work highlights the interplay of two main contexts of violence within Colombia that impacts on women’s well-being and resilience: cultural and political. Our recommendations work at those contextual levels, identifying methodologies—often social interventions—that would promote mental health competencies among women who work at levels of treatment and social change. For example, beyond the narrative therapy approaches suggested previously, given women’s connections to other women within families and communities, we suggest a community conversations approach to deepen solidarity and strengths and lay the foundations for social action on contexts of distress. For example, when used in a study related to improving maternal and child health in Zambia, women’s conversation groups developed activities linked to community sensitisation and building new infrastructure (toilets, health posts) to reduce poor health outcomes.^74^ The approach has also been used within mental health-related work among historically marginalised groups, such as Black African and Black Caribbean communities in the UK.^75^

The primary limitation to our study is its small sample size. However, this was overcome in two ways. First, through ensuring our sample and analysis was geared towards generating sufficient informational power^40^ to support our claims. The focused sample (all available women from our wider sample participated) and repeated interview sessions with each woman (two or more) meant that we have confidence that our themes are appropriately powered within this analysis. Second qualitative research has interests in generalisation that differ to quantitative research, namely through its ability to communicate alternative voices and truths, and overcome silencing within existing bodies of literature.^76^ As such, our work seeks to generalise as a form of problematisation, to make visible the knowledge of women’s survival through conflict, and its celebration of power and agency at work— confronting the cognitive empire of Eurocentric scholarship in this area, that often focuses on challenges people face, rather than the solutions already at work in their lives.^77^ Future studies should continue to explore these themes.

## Conclusion

Our work suggests that women are active leaders of their own lives, but also impacted by intersecting violence(s) and restrictive political economies that have gendered pathways to impact. Public mental health and government support programmes should be strength-based and build on women’s existing coping strategies, to ensure that programmes work alongside women’s own projects and hopes of survival. Interventions to improve mental health should particularly emphasise opportunities to promote redistribution of domestic work and childcare burden within families, as well promoting opportunities for the income generation alongside therapies. For women in situations of conflict mental health interventions which balance structural and relational supports should be mandatory, to overcome the false dichotomy between psychological and structural determinants of poor mental health.

## Data Availability

All data relevant to the study are included in the article or uploaded as supplementary information. Given the sensitive nature of the topics explored, this data is not available for public reuse, and all data relevant to this study are presented in a safe way with this article. For any questions relating to the data, please contact the corresponding author.
