# Supplementary material for: Gender, mental health and resilience in armed conflict: listening to life stories of internally displaced women in Colombia
Source: BMJ Glob Health. 2021 Oct 7;6(10):e005770. doi: 10.1136/bmjgh-2021-005770 (PMC8499256; doi:10.1136/bmjgh-2021-005770)
Supplement: Supplementary data [file bmjgh-2021-005770supp001.pdf]

Supplementary Data: Gender, mental health and resilience in armed conflict: Listening to Life stories of internally displaced women in Colombia

Section 1. Life history interview guide

Life History Interview Guide

1. What is your full name?
2. Did you have a nickname? How did you get it?
3. When and where were you born?
4. Tell me about your parents or your family background.
5. Where was your family originally from?
6. What did your parents do for a living?
7. Did you contribute to the family income or help your parents in their work in any way?
8. What was your parents' religious background?
9. How was religion observed in your home?
10. What were your parents' political beliefs?
11. Were they involved in any political organizations?
12. What other relatives did you have contact with growing up?
13. How many children were in the family, and where were you in the line-up?
14. Describe what your siblings were like. Who were you closest to?
15. Describe the house you grew up in. Describe your room.
16. What were your family's economic circumstances?
17. What were your duties around the house as a child?
18. When did you learn to cook and who taught you? Were there any special family foods or recipes?
19. What activities did the family do together?
20. What did you do on Christmas? Birthdays? Other holidays?
21. What is your earliest memory of home/family?
22. Tell me about the community you lived in
23. Describe your neighbourhood
24. Where did you go to elementary school?
25. What was school like for you?
26. What did you like about it? What was hard for you?
27. What did you do in your spare time?
28. Who were your best friends and what did you do when you got together?
29. Do you keep in touch with any of them?
30. Did you have any hobbies or special interests?
31. What did you want to be when you grew up?
32. How did your relationship with your parents change when you became a teenager? If you had conflict with them, what was it over?
33. Did you have chores around the house? What were they?

34. What were the different groups at your school?
35. Which did you belong to?
36. How do you think you were perceived by others?
37. Were you involved in any extracurricular activities?
38. What were they? What were your plans when you finished school? Education? Work?
39. What did your parents think of your plans?
40. What did your friends think? What did your friends plan to do?
41. Did the boys and girls in the family have different plans/expectations?
42. Did you have jobs during your teenage years? Doing what? Did you contribute to the family income? If not, how did you spend your money?
43. At what age did you begin dating? What kinds of activities did you do on dates?
44. Describe your first date.
45. What was the advice your parents gave you related to dating?
46. Did you get teaching on this in church or school? What was it?
47. What was your first job?
48. What kinds of jobs have you had?
49. How did you decide on your career?
50. How did the war change you?
51. How did you meet your husband?
52. What has been the most difficult thing about being married?
53. Describe the birth of your children
54. What were they each like when they were young?
55. How have they changed or not changed?
56. What were their relationships like with each other and with you when they were young? Now?
57. What activities did the family do together?
58. What family traditions did you try to establish?
59. Does your family have any heirlooms or objects of sentimental value? What is their origin, and how have they been passed down?
60. What was the most satisfying to you about raising children?
61. What was most difficult?
62. What values did you try to raise your children with?
63. How did you go about doing that?
64. What forms of discipline did you use and why?
65. How has being a parent changed you?
66. How has the war and the displacement changed you as a parent?
67. How was your family affected by the war?
68. How have you managed to stay afloat and provide your children with everything they need?

Section 2. Coding Framework: global, organising and basic themes

Table 1. Coding framework for womanhood and resilience in armed conflict: Life stories of displaced women in Colombia.

Global Themes	Organising Themes	Basic Themes	Examples of codes
Armed conflict and its gendered consequences on women´s emotional wellbeing	Adverse childhood experiences shaped by armed conflict and gender.	Abuse during childhood	Physical abuse by caregivers<br>Sexual abuse by nonfamily member<br>Psychological abuse by caregivers<br>Neglect during childhood
		Education restriction due to gender	Interruption of education because father wanted to protect from boys
		Absent parents	Interruption of education to take care of siblings
		Deprivation of childhood due to gender	Housework and take care of siblings
		Instability and migration	Migration<br>Raised by grandparents<br>Divorce or separation due to gender violence
		Threats and vulnerability in the community	Fear of recruitment to join the armed group<br>Too much violence in the neighbour to raise children<br>Threats to grandfather and torture
		Precipitating event or moment of departure	Murder of aunt<br>Kidnapping of husband
	Women's continuous and multicausal pathway of internal displacement	Search of a safer habitat	Moved to another town because family offered help<br>Search of a place without guerrillas' presence<br>Search for a calm environment to raise children
		Transition and adaptation	Difficulty adapting due to economic scarcity<br>Difficulty adapting because of lack of lack of governmental support<br>Good adaptation due to social support
		Multicausal displacement	Displacement due to gender violence<br>Displacement for education opportunity<br>Displacement due to better jobs opportunities<br>Displacement to take care of family
		Gender violence by armed groups	Sexual harassment, /intimidation by armed groups
	Intergenerational gender violence exacerbated by armed conflict	IPV towards themselves	Physical IPV
		IPV towards their daughters	Physical IPV<br>Psychological IPV<br>Economic IPV
		IPV towards their mothers	Physical IPV

			Psychological IPV
	Challenges of motherhood are made more hazardous by gender violence and the armed conflict.	Mother figure to siblings/family members	Adopting a mother role to their siblings to help mother<br>Help sister with childcare
		Mandatory motherhood	Unwanted pregnancy<br>Belief of motherhood inherent to women's nature<br>Became mother because of age<br>Partner insisted in pregnancy<br>Unequal childcare burden<br>Unable to work forced by partner
		IPV exacerbated during motherhood	Physical abuse<br>Hospitalization due to IPV
		High risk pregnancy due to armed conflict	Fear of pregnancy loss<br>Hospitalization due to complications in pregnancy.
		Self-destructive behaviours to cope	Alcohol consumption<br>Unsafe sex<br>Suicide Attempt
	Deterioration of women´s emotional wellbeing driven by the convergence of gender violence and armed conflict	Emotional distress associated with IPV	Nightmares and insomnia<br>Nervousness and anxiety<br>Sadness
		Emotional distress associated with the armed conflict	Nervousness<br>Fear of being shot
		Intergenerational emotional distress	Mother's addiction<br>Mother's sadness and loneliness<br>Mother's suicide<br>Father's alcoholism<br>Siblings'' drug addiction
Coping strategies co-occur with female empowerment	Women draw on power to exercise change	Cognitive power	Access to trainings and education<br>Desire to study and work<br>Breaking out with oppression and violence
		Bodied power	Taking care of their body and mind<br>Leaving abusive partner
		Social power	Being support to other women in need<br>Having support of their family to settle

		Support of community to be independent
		Support of family to leave abusive partner
		Motivation to work
		Started their own business
		Commercialization of food
		Commercialization of beauty product
	Material power	Work in bad conditions to have money
		Religious beliefs as an opportunity to cope
		Religion as spirituality
		Religion as a source of exclusion
	Symbolic power	Religion maintaining oppression and traditional roles
	Redistribution of gendered work	Teaching children to do housework<br>Demanding husband to do housework
		Access to economic aids by movement
		Elderly programs
		Women's networks maternity
	Social enablers in the wider society/ political economy	Cooking and entrepreneurship courses<br>Internally displaced status
	Social enablers in the community	Neighbours' support and sense of community.<br>Strong female relationships
Social enablers from different social environments		Family as a source of support: material resources
	Social enablers in the household	Family for emotional support<br>Mother's unconditional support

TABLE 1. PARTICIPANT'S DEMOGRAPHIC INFORMATION

PARTICIPANT # AND INITIAL	Age	Municipality of origin	Number of interview sessions
**1 MARÍA (L.C)**	59	Sucre	3
**2 LUCÍA (S)**	26	Sucre	3
**3 JOSEFINA (L)**	35	Tolima	1
**4 DORA (R)**	24	Cesar	3
**5 RIGOBERTA (Y)**	35	Santander	3
**6 SARA (D.R)**	73	Cundinamarca	3
**7 MIRIAM (S.P)**	Not reported	Córdoba	2

BMJ Publishing Group Limited (BMJ) disclaims all liability and responsibility arising from any reliance placed on this supplemental material which has been supplied by the author(s)
